# A New Form of Suture

**Published:** 1867-08

**Authors:** T. G. Comstock

**Affiliations:** Attending Physician St. Louis Good Samaritan Hospital


St. Louis, *June 8, 1867.*

Dr. J. Adams Allen, *Ed. Chicago Med. Journal:—*

*Dear Sir:*—I noticed in the number of your Journal for April and May last, an article written by Dr. Hoy, of Wisconsin, upon "A New Form of Suture."

The author of the article "calls the attention of the profession to it, not knowing that it is mentioned in any work upon surgery." This suture, which Dr. H. terms the "rubber-suture," is a thin, narrow ring of gum-elastic, and in his recommendation of its usefulness and practical value, I most heartily join; but he is in error, in supposing it is not mentioned in works upon surgery. Dr. W. L. Atlee, of Philadelphia, has used it for some years past in his operations for ovariotomy, an account of which may be found in the *American Journal of the Medical Sciences*, January, 1860, p. 81.

Dr. Gross, in his Surgery, third edition, vol. ii., page 342, speaks of India-rubber sutures, as having been first employed by one Mons. Rigal, and also by Dr. Atlee.

I have, for the past four years, occasionally employed these sutures in operations for hare-lip, but when used in this operation, I cannot say that they possess any merits over the twisted suture; in fact, in an operation for hare-lip, which I recently performed, I used the last named, in preference to the elastic suture. In cases where a great many sutures are requisite, and where they must be applied without delay, these India-rubber rings may be advantageously employed; and this is particularly the case in operations for ovariotomy. Whenever the surgeon is short of assistants in performing an operation, they will be found very handy. These gum-elastic rings are now for sale at most stationers, or they may be improvised by cutting off small sections, or rings, from ordinary India-rubber half-inch tubing. Yours, very respectfully,

T. G. COMSTOCK, M.D.,
*Attending Phys'n St. Louis Good Samaritan Hospital.*

